# Hydrothermal synthesis of Mn_3_O_4_ nanorods modified indium tin oxide electrode as an efficient nanocatalyst towards direct urea electrooxidation

**DOI:** 10.1371/journal.pone.0272586

**Published:** 2022-08-04

**Authors:** Waleed A. El-Said, Ahmad Alsulmi, Wael Alshitari

**Affiliations:** 1 Department of Chemistry, University of Jeddah, College of Science, Jeddah, Saudi Arabia; 2 Department of Chemistry, Faculty of Science, Assiut University, Assiut, Egypt; Minia University, EGYPT

## Abstract

Control fabrication of metal-oxide nanocatalysts for electrochemical reactions has received considerable research attention. Here, manganese oxide (Mn_3_O_4_) nanorods modified indium tin oxide (ITO) electrodes were prepared based on the in-situ one-step hydrothermal methods. The nanorods were well characterized using field emission scanning electron microscopy, Fourier transform infrared, and X-ray diffraction spectroscopy. The results showed the formation of pure crystalline Mn_3_O_4_ nanorods with a length of approximately 1.4 μm and a thickness of approximately 100 ± 30 nm. The Mn_3_O_4_ nanorod-modified ITO electrodes were used for accelerating urea electrochemical oxidation at room temperature using cyclic and square wave voltammetry techniques. The results indicated that the modified electrode demonstrated excellent electrocatalytic performance toward urea electrooxidation in an alkaline medium over concentrations ranging from 0.2 to 4 mol/L. The modified electrode showed high durability, attaining more than 88% of its baseline performance after 150 cycles; furthermore, the chronoamperometry technique demonstrated high stability. Thus, the Mn_3_O_4_ nanorod-modified ITO electrode is a promising anode for direct urea fuel cell applications.

## 1. Introduction

While fossil fuel as an energy source is an important pillar of global economic development, it does not promote human existence because of its associated high environmental pollution **[1]**. Consequently, searching for alternative and sustainable energy sources has become a global goal to maintain current energy consumption and prevent environmental catastrophes **[1–7]**. Owing to the several advantages of the fuel cells including their environmentally friendly, and high efficiency. Thus, fuel cells are among the uppermost other renewable sources, including solar, wind, and hydropower energies **[7–9]**. Hydrogen is considered the future fuel of our economy and life **[7]**. However, many challenges have evolved in hydrogen production, storage, and transportation **[1]**. Accordingly, several liquid fuels, including methanol, ethanol, and formic acid, have been used for electricity production, with significant advantages **[10]**. However, the use of these fuels has increased carbon emissions. Thus, nitrogen-based fuels such as ammonia and hydrazine have been used to generate hydrogen gas using thermal, catalytic, or electrolytic methods, which are promising carbon-free energy sources **[11, 12]**. Furthermore, N-based fuels are directly used to produce electricity without any preconversion **[13, 14]**. Although, liquid fuels have advantages owing to their high energy density and convenience to stockpile at low costs compared with gaseous fuels. However, liquid fuels suffer many challenges regarding safety, including toxicity **[15]** and volatilization. Thus, the use of solid fuels with high energy densities could be a potential solution to these challenges **[16, 17]**.

Urea is a well-known fertilizer **[8, 18, 19]**, with a considerable energy density of 16.9 MJ/L, which is 10 times greater than that of hydrogen **[8, 18]**. Green hydrogen is produced by water electrolysis, requiring an overall cell voltage of 1.23 V **[20]**. While green hydrogen production based on electrolyzed urea needs only 0.082 V, saving energy and cost **[20]**, urea produces 10 wt% valid hydrogens.

The uses of urea as a solid fuel have additional advantages, including (1) high energy density; (2) high solubility; (3) the manufacturing of urea, including the use of CO_2_, making it a CO_2_-neutral energy source; (4) a solution for environmental issues resulting from the use of urea as a fertilizer or urine; (5) a potential solution for treated nitrogen-rich wastewater using urine as a direct fuel; (6) high stability; (7) relatively non-toxic; (8) a safe and easily transported carrier; and (9) low cost. These advantages promote urea as the best sustainable hydrogen carrier and provide a sustainable energy supply **[21–24]**.

Industrial and municipal wastewater, especially those containing urea, must be treated for environmental protection and energy production **[8, 9]**. Thus, urea can be electrooxidized to generate electricity while purifying wastewater simultaneously **[7, 8, 24]**.

The first direct urea fuel cell (DUFC) was developed in 2010 **[25]**. Alkaline urea electrooxidation has been reported as the best method for urea-containing wastewater treatment and urea electricity generation **[23]**. The electrooxidation of urea in an alkaline medium has been shown in Eqs (**1–3**) **[26]**. Typically, electrooxidation of the fuel (urea) includes a reaction with the supporting electrolyte (KOH) in certain proportions. Two reaction mechanisms based on the use of urea have been proposed: (i) the electrochemical reaction when urea is applied as a fuel for DUFC, as shown in Eqs (**1–3**), and (ii) the use of urea for the electrolytic production of hydrogen, as shown in Eqs (**4–6**). The anodic and cathodic reactions of urea indicated that the molar ratio of KOH should be approximately 8 or 6 times that of urea **[27]**.


$$Anodic\;reaction:\;CO{\left(NH_{2}\right)}_{2}+8OH^{-}‐‐‐‐\to \;N_{2}+6H_{2}O+CO_{3}{}^{2-}+6e^{-}$$ (1)

$$Cathodic\;reaction:\;O_{2}+2H_{2}O+4e^{-}‐‐‐‐\to \;4OH^{‐}$$ (2)

$$Overall\;reaction:\;CO{\left(NH_{2}\right)}_{2}+O_{2}+4OH^{‐}‐‐‐‐\to \;CO_{3}{}^{2-}+N_{2}+4H_{2}O$$ (3)

$$Anodic\;reaction:\;CO{\left(NH_{2}\right)}_{2}+8OH^{-}‐‐‐‐\to \;N_{2}+6H_{2}O+CO_{3}{}^{2-}+6e^{-}$$ (4)

$$Cathodic\;reaction:\;2H_{2}O+2e^{-}‐‐‐‐\to \;2OH^{-}+H_{2}$$ (5)

$$Overall\;reaction:\;CO{\left(NH_{2}\right)}_{2}+6OH^{-}‐‐‐‐\to \;CO_{3}{}^{2-}+N_{2}+4H_{2}O$$ (6)

Metallic nickel (Ni) is considered a super-effective non-precious catalyst that is nearly becoming the standard material in DUFC production **[1, 7, 18, 20, 22–24]**. Nevertheless, carbon monoxide (CO) can easily contaminate pure Ni and reduce its electroactive sites **[23]**. The Ni poisoning could be solved by combining Ni with other metal and metal oxides (e.g., Co_3_O_4_ and Ni-Mo) **[8, 23, 28]**, or carbonaceous materials (graphene, or polymers) **[18, 23, 24]**. Recently, several metal oxide nanomaterials were reported as electrocatalytic materials for enhancing energy storage and production **[29–32]**. To reduce the cost of fuel cells, research has focused on developing transition metal-modified electrodes as anodes for the direct oxidation of urea fuel.

Here, we fabricated Mn_3_O_4_ nanorod-modified ITO electrodes based on the hydrothermal process. The modified electrode was used to develop DUFCs. The fabricated Mn_3_O_4_ nanostructures exhibited uniform three-dimensional nanorods with a length of approximately 1.4 μm and a thickness of approximately 100 ± 30 nm. The electrocatalytic performance of the Mn_3_O_4_ nanorod-modified ITO electrode for urea electrooxidation was investigated using CV and SWV techniques at room temperature over a wide range of concentrations, from 0.2 to 4 mol/L. Moreover, the Mn_3_O_4_ nanorod-modified ITO electrode has high durability, making it a promising anode for DUFC applications.

## 2. Materials and methods

### 2.1. Materials

Manganese sulfate monohydrate (MnSO_4_.H_2_O) and hydrogen peroxide (H_2_O_2_) were purchased from Panreac (Barcelona, Spain). Urea, ammonia solution, and potassium hydroxide (KOH) were obtained from Sigma-Aldrich. Deionized water (DIW) was used to prepare all aqueous solutions.

### 2.2. Fabrication of the Mn_3_O_4_ nanorods-modified ITO electrode

Manganese oxide nanoparticles (NPs) were formed through the hydrothermal process in the presence of H_2_O_2_ and KMnO_4_. Typically, 1.69 g of MnSO_4_, 3.1 g of KMnO_4_, and 35 mL of H_2_O_2_ were dissolved together in 40-mL DIW. The formed solution was kept under stirring for 30 min. The ITO electrodes (1 × 2 cm) were cleaned using a basic Piranha solution, DIW, and ethanol, and then dried **[29–31]**. The solution was then transferred into the autoclave vessel, and the ITO electrodes were inserted horizontally inside the reaction mixture. The autoclave was closed tightly and kept in an oven at 80°C for 2 hours. The modified electrodes were cleaned with DIW and ethanol and then dried in an oven at 80°C.

### 2.3. Instruments

The Fourier transform infrared (FTIR) spectrum of the manganese oxide NPs was studied with a Nicolet 6700 Thermo Fisher Scientific spectrophotometer using the KBr pellet technique. The morphology of the manganese oxide NPs was investigated using the scanning electron microscopy (SEM) technique with a Jeol JSM-5400 LV instrument. Furthermore, the X-ray diffraction (XRD) of the manganese oxide sample was studied using a Philips X-ray PW 1710 diffractometer and Ni-filtered CuKa radiation.

## 3. Results and discussion

### 3.1. Characterization of the Mn_3_O_4_-modified ITO electrode

**Fig 1:**
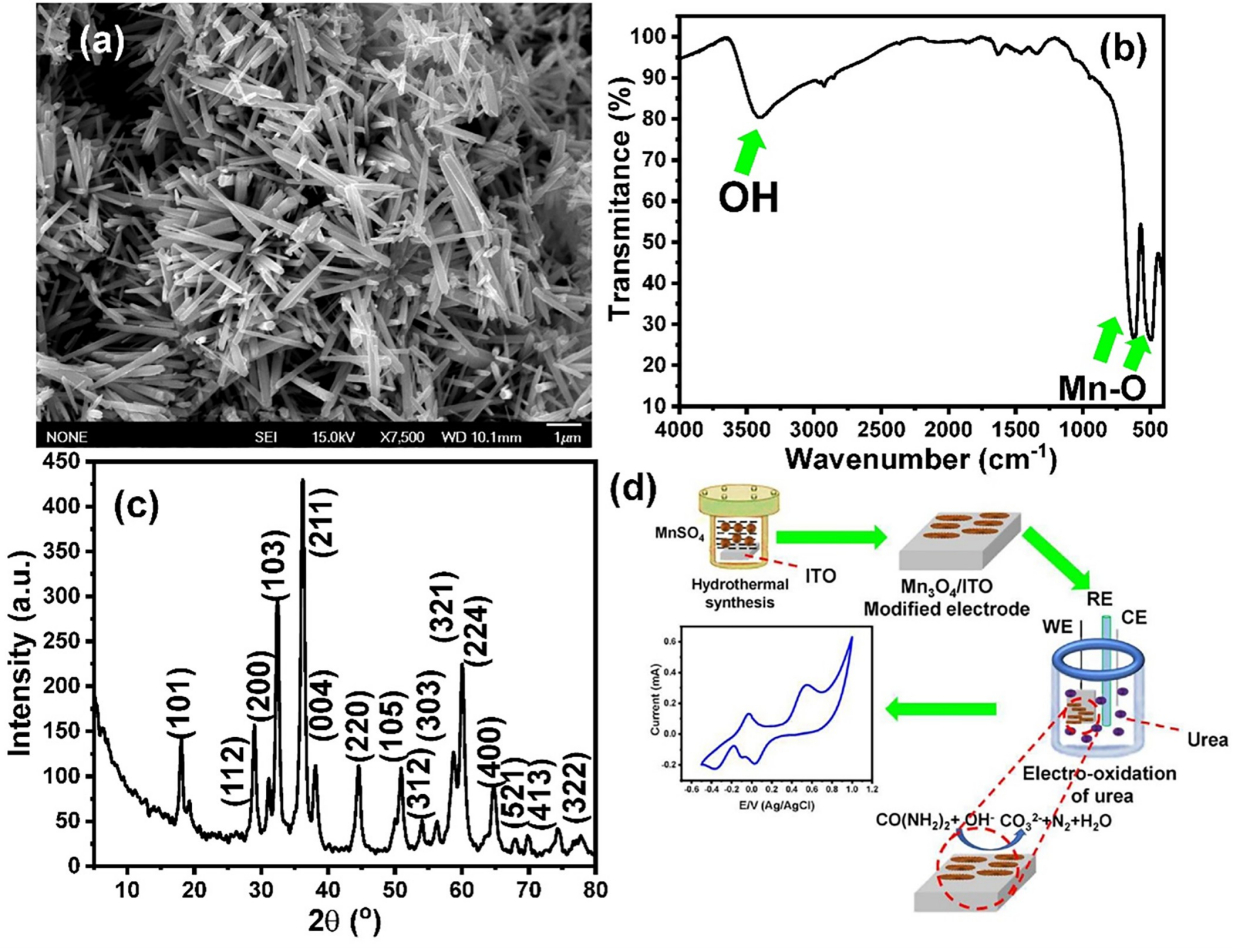
(a) SEM image of the Mn_3_O_4_ nanorods, (b) FTIR spectrum of the as-prepared Mn_3_O_4_ nanorods, (c) XRD pattern of the as-prepared Mn_3_O_4_ nanorods, and (d) schematic diagram for the development of the direct urea fuel cell based on Mn_3_O_4_ nanorods modified ITO electrode.

The morphology of the prepared manganese oxide was investigated using the SEM technique. The SEM image of the prepared manganese oxide (**Fig 1A**) depicts the formation of nanorod structures. The results showed the formation of uniform nanorods with an average thickness of 100 ± 30 nm and a length of 1.4 μm.

**Fig 1B** shows the FTIR spectrum of the as-prepared manganese oxide nanoparticles (NPs). The FTIR results showed characteristic bands at 483 and 633 cm^−1^ for the stretching vibration of Mn-O and Mn-O-Mn. On the other hand, the weak band at 1630 cm^−1^ and broadband at 3430 cm^−1^ are attributed to the OH group of the adsorbed water molecules. These FTIR data are in good agreement with previously reported data on manganese oxide NPs **[32, 33]**.

The phases and oxidation states of the manganese oxide NPs were investigated using XRD. **Fig 1C** shows the XRD pattern of the as-prepared manganese oxide material and sharp diffraction peaks with high intensities, indicating the formation of a nanocrystalline product. The XRD pattern showed a set of diffraction peaks at 18.04° (101), 29.02° (112), 31.12° (200), 32.5° (103), 36.16° (211), 38.14° (004), 44.62° (220), 50.92° (105), 54.1° (312), 56.26° (303), 58.72° (321), 60.04° (224), 64.78° (400), 69.88° (521), 74.32° (413), and 77.8° (332). These diffraction peaks are consistent with those of the hausmannite Mn_3_O_4_ NPs (JCPDS: No. 24–0734) **[33–39]**.

The average crystallite size (D) of the Mn_3_O_4_ nanorods was calculated using Debye Scherer’s formula, as expressed in Eq (7) **[40–42]**. The average crystallite size was obtained based on the width of the peaks (310, 103, 211, 004, 220, and 224). The crystallite size of the as-prepared Mn_3_O_4_ nanostructures was 26 ± 3 nm.

$$D=\frac{K\lambda }{\beta cos\theta }$$ (7)

where *K* is the particle shape factor (≈0.9), *λ* is the X-ray wavelength (= 1.541838), *β* is the full width at half-maximum, and *θ* is the diffraction angle.

### 3.2. Direct electrooxidation of urea

**Fig 2:**
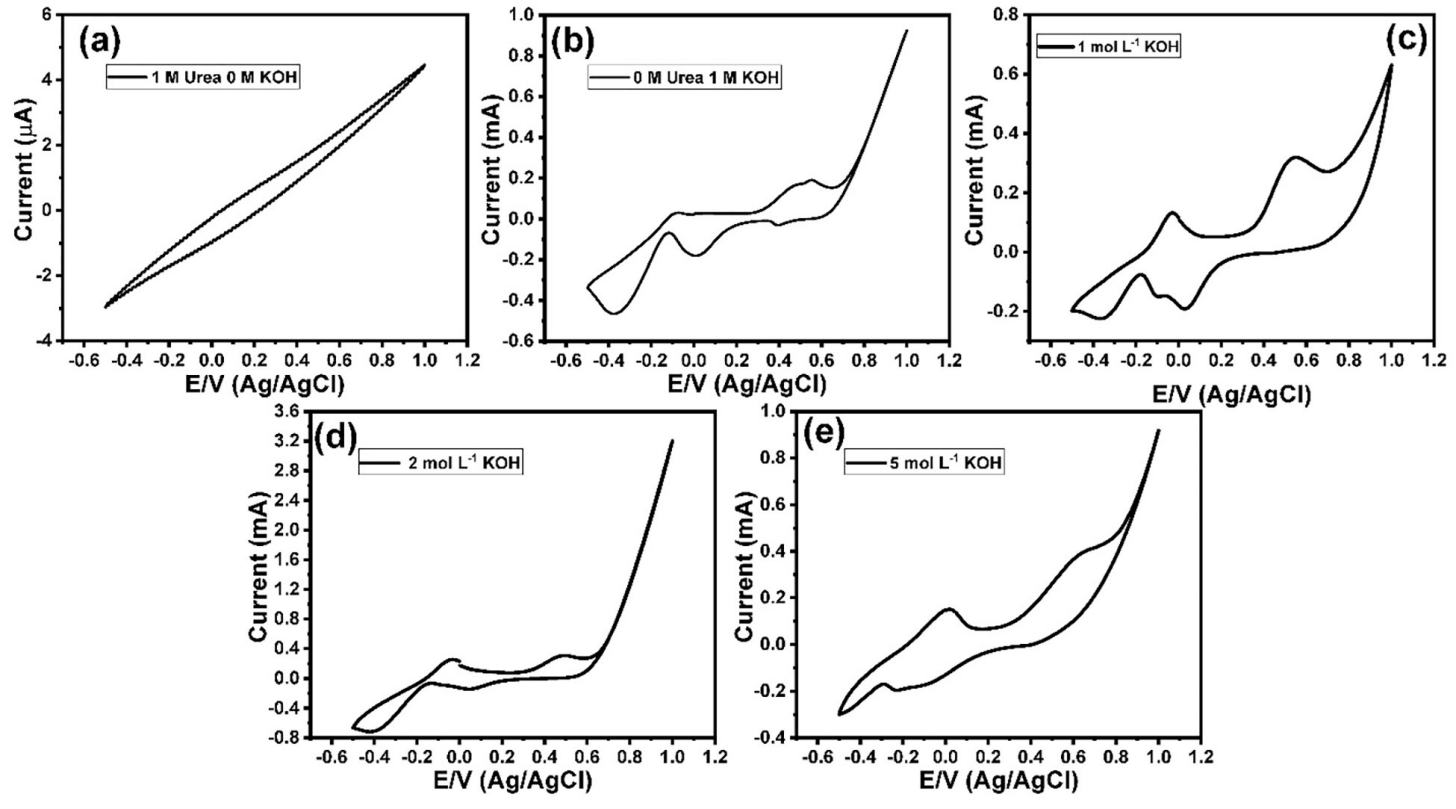
Cyclic voltammograms of (a) Mn_3_O_4_ nanorods modified ITO electrode in the presence of 1 mol L^-1^ of KOH, (b) Mn_3_O_4_ nanorods modified ITO electrode in the presence of 1 mol L^-1^ of urea in the absence of KOH, (c) Mn_3_O_4_ nanorods modified ITO electrode in the presence of 1 mol L^-1^ of urea and 1 mol L^-1^ of KOH, (d) Mn_3_O_4_ nanorods modified ITO electrode in the presence of 1 mol L^-1^ of urea and 2 mol L^-1^ of KOH, and (e) Mn_3_O_4_ nanorods modified ITO electrode in the presence of 1 mol L^-1^ of urea and 5 mol L^-1^ of KOH. Scan rate 100 mV/s.

The fabricated Mn_3_O_4_ nanorods/ITO electrodes were used as a working electrode for developing a direct urea fuel cell, as shown in **Fig 1D**. To investigate the electrooxidation of urea at the Mn_3_O_4_ nanorods/ITO electrodes and to optimize the electrooxidation conditions, the effects of the pH values and the scan rate on the urea electrooxidation were investigated. **Fig 2A** shows the cyclic voltammetry (CV) response of 1 mol/L of urea in the absence of OH^−^, in which no redox peaks could be observed. The CV of the Mn_3_O_4_ nanorods/ITO electrodes in 1 mol/L of KOH showed two oxidation peaks at 0.485 and 0.555 V, and two reduction peaks at −0.18 and −0.465 V (**Fig 2B**). These two reduction peaks could be attributed to the formation of MnOOH and Mn(II), respectively, which is in good agreement with previously reported data **[43]**. **Fig 2C** shows the electrooxidation of 1 mol/L urea in the presence of 1 mol/L of KOH, depicting a couple of redox peaks, including an oxidation peak at −0.02 V and a cathodic peak at −0.07. Moreover, the onset potential was found to be at -0.21 V (**Fig 2C**). The effects of different pH values on the CV response of urea at the Mn_3_O_4_ nanorods/ITO electrodes were also investigated (**Fig 2D and 2E**). The results indicated the shifting of the redox peaks toward the negative direction with increasing pH values. This shifting to the negative direction suggests that this electrooxidation process is thermodynamically more favorable at higher KOH concentrations **[23]**.

**Fig 3:**
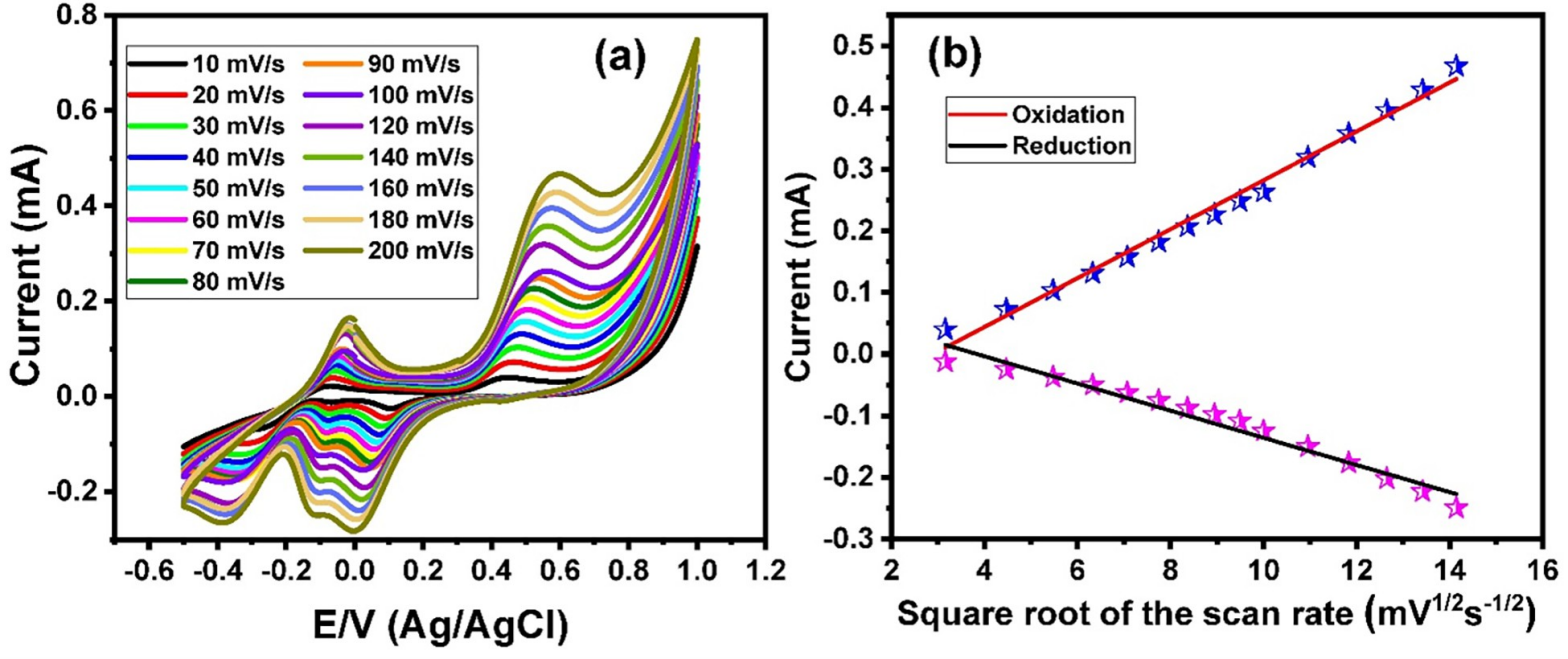
(a) Cyclic voltammograms of 1 mol L^-1^ of urea in the presence of 1 mol L^-1^ of KOH at Mn_3_O_4_ nanorods modified ITO electrode under different scan rates within a range from 10 mV/s to 200 mV/s, and (b) the relationship between the redox current values and the square root of the scan rate.

The effect of the scan rate on the electrooxidation of urea at the Mn_3_O_4_ nanorods/ITO electrodes was investigated. **Fig 3A** shows the cyclic voltammograms of 1 mol/L at the Mn_3_O_4_ nanorods/ITO electrodes under different scan rates ranging from 10 to 200 mV/s, which showed increasing redox current peaks with increasing scan rates. The relationship between the square root of the scan rates and the oxidation current peak showed a linear curve (**Fig 3B**). These results demonstrate that the electrooxidation of urea at Mn_3_O_4_ nanorods/ITO electrodes is a revisable process.

Based on these results, all electrooxidation measurements were performed in the presence of 1 mol/L KOH at a scan rate of 100 mV/s. The performances of the developed electrode toward the electrooxidation of urea in comparison with several reported electrodes were summarized in **Table 1**. It is worth noting that the developed modified electrode has a lower onset potential compared with many of the reported electrodes **[44–61]**. The lower oxidation onset potential of the Mn_3_O_4_ nanorods/ITO electrode indicated its capability to work in a real fuel cell **[28]**.

**Table 1:** Activity of different electrodes towards urea electrooxidation vs Ag/AgCl.

Electrode	Onset potential (V)	Anodic peak potential (V)	Scan rate (mV/s)	Ref.
NiO/Gr-200	-0.36	0.51	20	45
NiO-Fe_2_O_3_/rGO/PVA	-0.36	0.51	20	46
NiMn/C, 90 wt%Ni	-0.085	-0.58	50	47
Nickel nanowire arrays	0.25	0.5	10	48
Nickel NWA	-0.1	0.45	10	49
NiPO	0.33	0.65	50	50
NiO/Gt	0.345	-0.62	10	51
Ni-loaded Gr	-0.38	-0.75	50	52
NiCr/C, 40%Cr	0.32	0.55	10	53
Co_7_Ni_3_-hcp	0.314	0.5	50	54
NiO/Gt-15	0.345	0.64	10	55
Ni_5_Cd_5_/CNF	0.35	0.67	50	56
Ni_0.9_Co_0.1_/CNF	0.32	0.86	50	57
NiMn/CNF	0.29	0.58	50	58
Ni/NeCNF*	0.36	0.73	50	59
Ni_x_Co_3-x_O_4_	0.28	0.5	10	60
Ni_1.5_Mn_1.5_O_4_	0.29	0.58	10	61
Ni(OH)_2_/NF	0.21	0.56	10	62
Mn_3_O_4_ nanorods/ITO	-0.21	-0.02	100	Current work

### 3.3. Performance of the Mn_3_O_4_ nanorods/ITO electrodes for the electrocatalytic oxidation of urea

**Fig 4:**
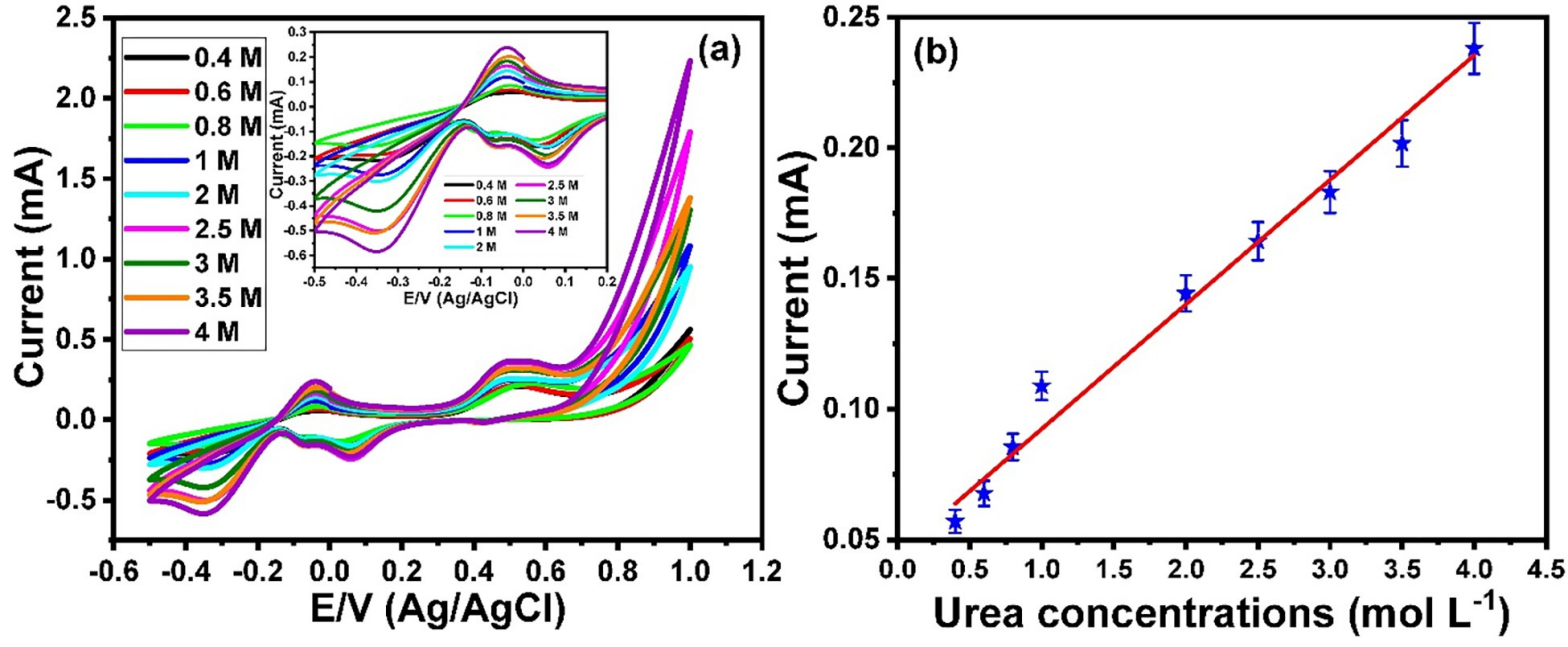
(a) Cyclic voltammograms of different concentrations of urea within a range from 0.4 mol L^-1^ to 4 mol L^-1^ in the presence of 1 mol L^-1^ of KOH at Mn_3_O_4_ nanorods modified ITO electrode (potential window from -0.5 V to 1 V), inset Square wave voltammograms of different concentrations of urea within a range from 0.4 mol L^-1^ to 4 mol L^-1^ in the presence of 1 mol L^-1^ of KOH at Mn_3_O_4_ nanorods modified ITO electrode (potential window from -0.5 V to 0.2 V), and (b) the relationship between the urea concentrations and oxidation current peaks. Scan rate 100 mV/s against Ag/AgCl electrode.

To investigate the performance of the Mn_3_O_4_ nanorods/ITO electrodes toward direct urea electrooxidation in the presence of 1.0 mol/L KOH, the influence of urea concentrations on catalytic activity was examined. **Fig 4A** shows the cyclic voltammetry voltammograms of different urea concentrations within the range of 0.4 to 4 mol/L over a potential window from −0.5 to 1 V. The inset in **Fig 4A** shows the voltammograms over a potential range of −0.5 to 0.2 V. The results showed that the redox current peak values gradually increased with increasing urea concentration. The relationship between the urea concentrations and the oxidation current peak is represented in **Fig 4B**, which illustrates a linear curve within a wide range of 0.2 to 4 mol/L.

**Fig 5:**
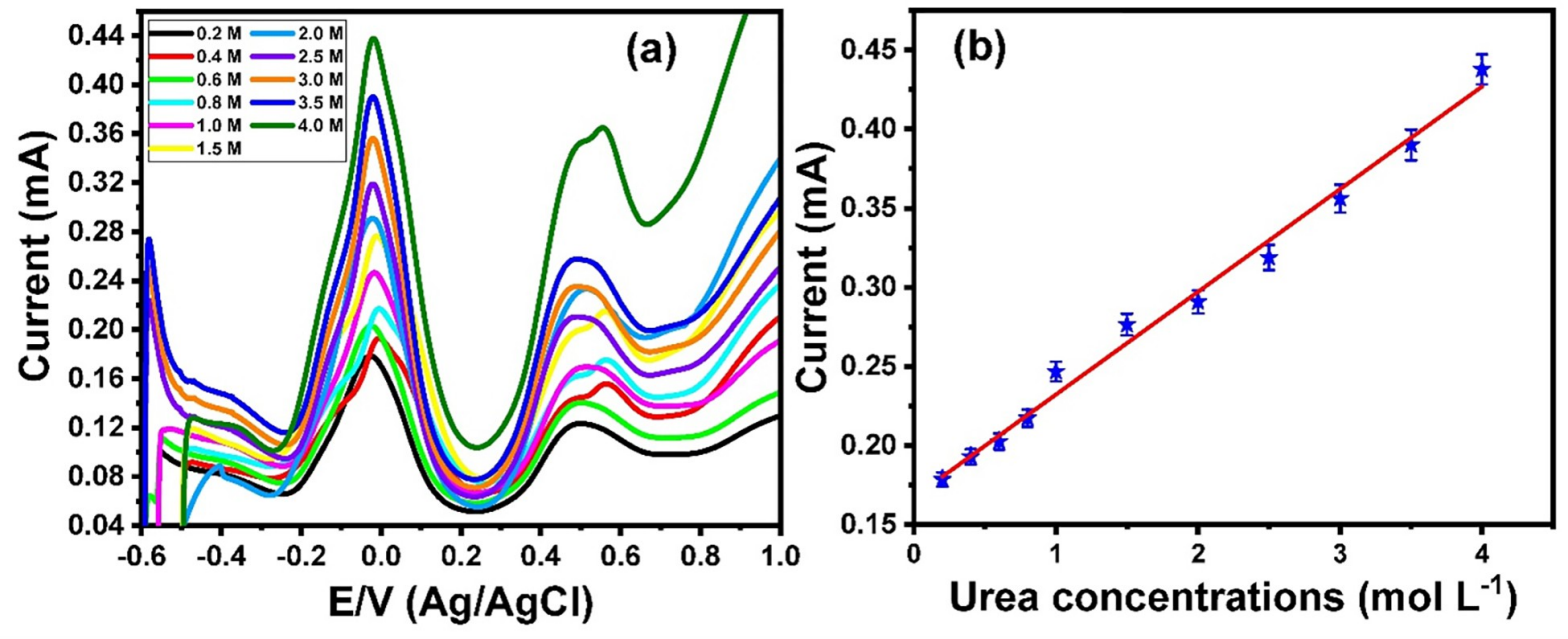
(a) Square wave voltammograms of different concentrations of urea within a range from 0.4 mol L^-1^ to 5 mol L^-1^ in the presence of 1 mol L^-1^ of KOH at Mn_3_O_4_ nanorods modified ITO electrode, and (b) the relationship between the urea concentrations and oxidation current peaks. Scan rate 100 mV/s against Ag/AgCl electrode.

Furthermore, the square wave voltammetry technique was used to examine the electrooxidation of urea in Mn_3_O_4_ nanorod-modified ITO electrodes. **Fig 5A** represents the square wave voltammograms for the electrooxidation of a wide range of urea concentrations from 0.2 to 4 mol/L over a potential range of −0.6 to 1 V. The data showed an oxidation peak at approximately −0.02 V, and the oxidation current peak increased with the increasing urea concentration. The relationship between urea concentrations and oxidation current peaks is demonstrated in **Fig 5B**, which illustrates a linear curve.

**Fig 6:**
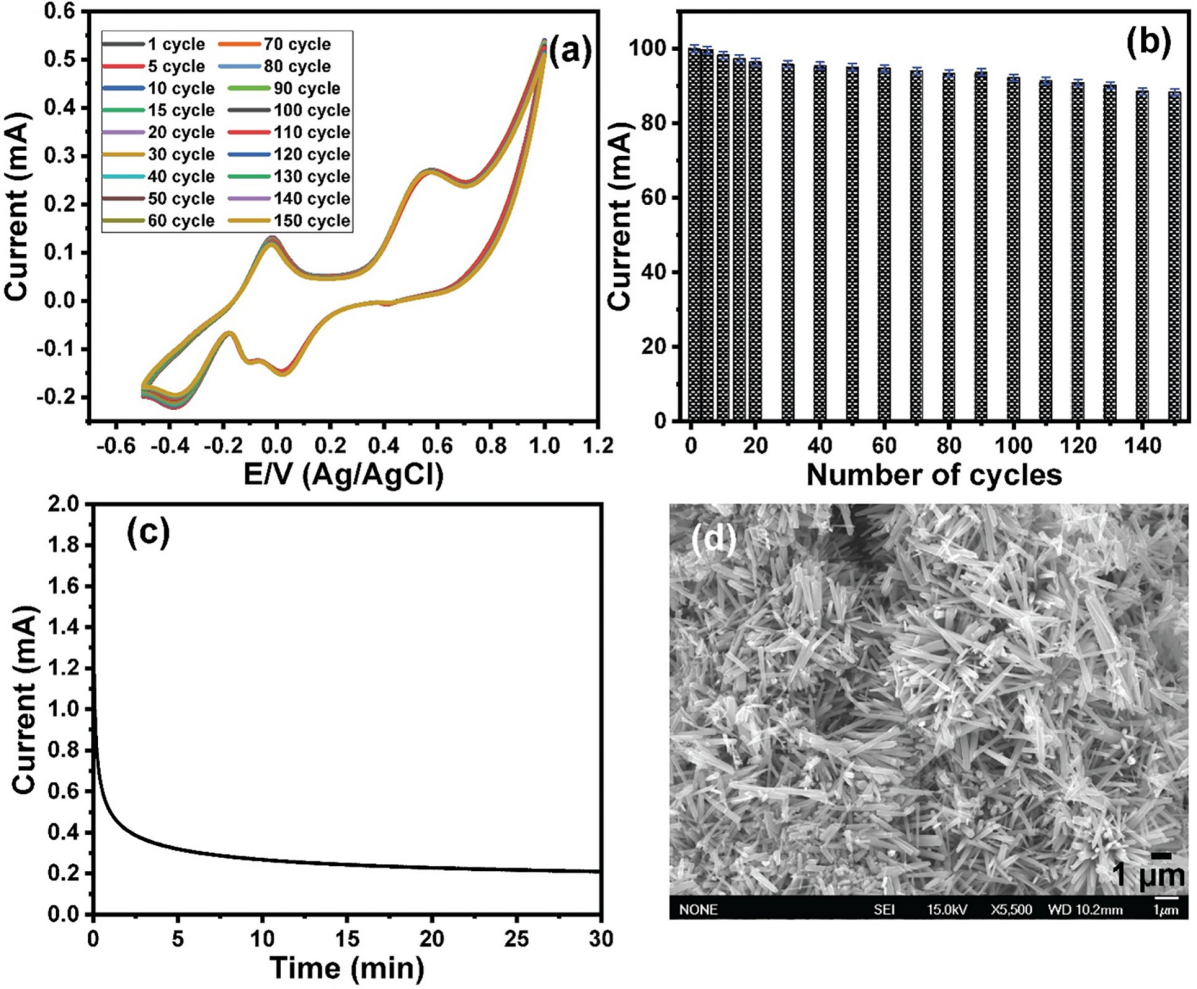
(a) Cyclic voltammograms of 1 mol L^-1^ of urea in the presence of 1 mol L^-1^ of KOH at Mn_3_O_4_ nanorods modified ITO electrode over 150 cycles, (b) the relationship between the oxidation current peaks and the cycle number. Scan rate 100 mV/s against Ag/AgCl electrode, (c) the chronoamperometry response of 1 mol L^-1^ of urea in the presence of 1 mol L^-1^ of KOH over 30 min, and (d) SEM image of the modified ITO electrode after its uses for electrooxidation of urea.

The durability of the Mn_3_O_4_ nanorod-modified ITO electrode was investigated by monitoring the changes in redox current peaks over 150 cycles. **Fig 6A** shows the cyclic voltammetry voltammograms of 1 mol/L urea in the presence of 1 mol/L KOH over 150 cycles. As can be seen in the figure, the redox current peaks slightly decreased after 150 cycles. The effect of the number of cycles on the oxidation is shown in **Fig 6B**, which demonstrates that the oxidation current decreased by approximately 12% of its efficiency after 150 cycles. These results demonstrate the high stability of the developed electrode after 150 cycles. Furthermore, the stability of the modified electrode was investigated based on the chronoamperometry technique. **Fig 6C** showed the chronoamperometry of the Mn_3_O_4_ nanorods modified ITO electrode for 30 min. The test was performed at a potential of 0.1 V vs. Ag/AgCl for 30 min in 0.5 mol/L KOH containing 0.5 mol/L of urea. The results showed the appearance of a continuous, stable, and high current density. It is worthwhile to note that the resulting current was slightly decreased with time. This current decrease is attributed to the consumption of electroactive species (i.e., urea) close to the surface of the modified electrode, besides the adsorption of the gaseous byproducts onto the nanocatalyst, which reduced the catalyst efficiency **[62]**. Thus, the Mn_3_O_4_ nanorods/ITO electrodes are stable and active electrodes toward direct urea electrooxidation. Moreover, the stability of the catalyst over the electrode was studied by investigating the morphology of the modified electrode after its use. **Fig 6D** showed the SEM image of the Mn_3_O_4_ nanorods modified ITO after 100 cycles of its uses for electrooxidation of urea. The results showed that the modified electrode has the same nanorod morphology as that of the as-prepared modified electrode, which indicated the stability of the Mn_3_O_4_ nanorods nanocatalyst over the ITO electrode surface.

## 4. Conclusion

A simple, in-situ, and one-step hydrothermal-based method was used for direct depositing Mn_3_O_4_ nanorod onto the ITO electrode surface. The results showed the formation of Mn_3_O_4_ nanorods with a well-aligned three-dimensional structure with a length of approximately 1.4 μm and a thickness of approximately 100 ± 30 nm. The Mn_3_O_4_ nanorod-modified ITO electrode demonstrated excellent electrocatalytic performance toward urea electrooxidation, resulting in a high current peak density at a urea concentration of 4 mol/L at room temperature. Furthermore, the cyclic voltammetry and chronoamperometry results demonstrated the high stability of the modified electrode.
